# View-Invariant Visuomotor Processing in Computational Mirror Neuron System for Humanoid

**DOI:** 10.1371/journal.pone.0152003

**Published:** 2016-03-21

**Authors:** Farhan Dawood, Chu Kiong Loo

**Affiliations:** Department of Artificial Intelligence, University of Malaya, Kuala Lumpur, Malaysia; Plymouth University, UNITED KINGDOM

## Abstract

Mirror neurons are visuo-motor neurons found in primates and thought to be significant for imitation learning. The proposition that mirror neurons result from associative learning while the neonate observes his own actions has received noteworthy empirical support. Self-exploration is regarded as a procedure by which infants become perceptually observant to their own body and engage in a perceptual communication with themselves. We assume that crude sense of self is the prerequisite for social interaction. However, the contribution of mirror neurons in encoding the perspective from which the motor acts of others are seen have not been addressed in relation to humanoid robots. In this paper we present a computational model for development of mirror neuron system for humanoid based on the hypothesis that infants acquire MNS by sensorimotor associative learning through self-exploration capable of sustaining early imitation skills. The purpose of our proposed model is to take into account the view-dependency of neurons as a probable outcome of the associative connectivity between motor and visual information. In our experiment, a humanoid robot stands in front of a mirror (represented through self-image using camera) in order to obtain the associative relationship between his own motor generated actions and his own visual body-image. In the learning process the network first forms mapping from each motor representation onto visual representation from the self-exploratory perspective. Afterwards, the representation of the motor commands is learned to be associated with all possible visual perspectives. The complete architecture was evaluated by simulation experiments performed on DARwIn-OP humanoid robot.

## Introduction

**Fig 1:**
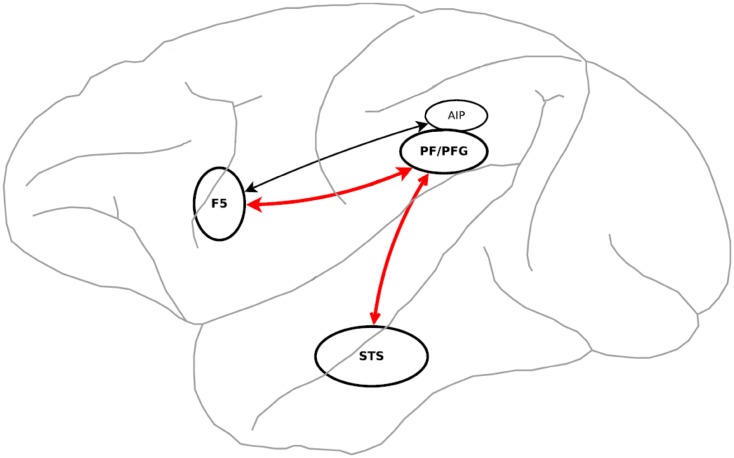
Schematic illustration of MNS in the macaque monkey brain. Area F5 is connected with the inferior parietal lobule (areas AIP-anterior intra-parietal, PF and PFG). Within the frontal lobe, area F5 is connected with hand/mouth representations of primary motor cortex (Source: Craighero et al. [16]).

Mirror Neurons belongs to the family of visuomotor neurons which were originally discovered in the F5 area located in the premotor cortex of the macaque monkey brain [1]. Mirror neurons not only activate when the primates observes an action (e.g., grasping) performed by demonstrator (human or monkey) and also activate when the primates try to execute the same observed action [2–4]. These neurons are an example of a motor resonance system in which brain activity pertaining to both observation and execution of action have an influence on each other. The discovery of mirror neuron and their functional hypothesis presented in the literature suggests that mirror neurons form the foundation of action understanding [1, 5], motion imitation [6, 7] and language development [8]. Rizzolatti & Sinigaglia [9] summarized mirror neuron function as ascribed to the “*parieto-frontal action-observation action-execution brain circuit*” or the mirror neuron system (MNS). The schematic interpretation of the mirror neuron system and its relevant circuitry connections are depicted in Fig 1. The mirror neuron system comprises of the area F5 [1, 2], area Parieto Frontal Gyrus (PFG) in the rostral part of the Inferior Parietal Lobule (IPL) between areas PF and PG [10, 11], Superior Temporal Sulcus (STS) [12, 13] and the Anterior Intraparietal area (AIP) [2]. Both parietal areas are attached with F5 and both receive visual information from areas located inside the STS providing input to frontal motor-control area F5. Similar to F5 region, STS encodes motion, however, it is derived of motor properties and consequently cannot be regarded as a true part of the mirror neuron system.

STS plays an important role in action understanding and mirror neuron system functionality. Perrett et al. [14] investigated that neurons in STS responds selectively to different perspectives of an object or action. These view-invariant or object-centered neurons have distinct anatomical position inside the STS. Neurons discerning to the viewpoint are located in posterior areas of STS (STSp) and the neurons related to view-invariant are positioned in anterior areas of STS (STSa), which are adjacent to frontal cortices [15]. STS area is linked with F5 via two different pathways [13]. The posterior part of STS (STSp) is linked with F5c via PF pathway (PFG) and the anterior part of STS (STSa) is linked with F5a via AIP.

For the development of mirror neurons, it has been theorized that they are a by-product of associative learning: when the organism generates motor commands the internal representation of this command and it’s observed visual effects are associated inside the mirror neuron system. Later, when the system is demonstrated with a visual stimulus that corresponds to one of the stored patterns, the associated motor command representation is triggered and retrieved automatically. This “Associative Sequence Learning” [17, 18] proposes that mirror neuron is shaped through sensorimotor experience. The associative hypothesis implies that the perceptual-motor coupling properties of mirror mechanism results from an associative learning process [19].

Functional properties of mirror neurons suggest that they are an important part of imitation learning. Many neuroscientists regard imitation as mediated by mirror neurons in humans [20]. Most developmental theories emphasize that social interactions, in particular understanding of actions, could be first achieved through imitation. However, the discussion on the origin of primitive imitative abilities is often neglected, referring instead to the possibility of its innateness [21, 22]. It has been contended, both in theory and experiments, that elementary forms of imitation could emerge as a result of self-exploration [23]. According to this hypothesis, a primitive sense of self is prerequisite for successful social interaction rather than an outcome of it. From birth, infants are not only involved in recognizing themselves during the process of perceiving environment, but they also exhibit exploratory commotion that appears to be particularly pointed towards the discovery of their own body’s attributes [24, 25]. They start developing associations between their motor commands and their resulting perceptual effects. The ability to imitate builds upon the development of visuo-motor contingencies, and self-concept of one’s own body’s constraints and capabilities [26]. In this view, infants have a proprioceptive sense of self that derives in part from their own body movements which the authors [24] have called ’body babbling’. Body babbling is the process of learning how specific body parts achieve various elementary configurations.

A fundamental question in developmental psychology is that how human infants develop a sense of self [27]. Piaget [28] contended that neonates learn to associate self and other through mirror play and tactual exploration of their own observed faces. Lewis et al. [29] suggested that human infants seem to become self-aware when they begin to recognize and discern themselves in a mirror. Physiological experiments [30–32] show that these observations provide a link between mirror image and self-awareness. To assess the mirror self-awareness, Gallup [30] and Amsterdam [33] applied a tool referred to as mirror test. They have discovered that animals and infants observe their own body movements in front of the mirror to explore specific kinaesthetic-visual egression of their action consequences. The mirror test not only plays an essential role in the analysis of animal behaviour, but it also reveals insight into the development of self-awareness in humans.

A robot can be more than a passive observer of the world as it learns and develops. Applying the approach, derived from the way human beings learn, will greatly enhance the usefulness and ability of robots in the human environment. Thus, our computational model for development of MNS is based on the hypothesis that: infants acquire MNS as induced by acquisition of sensory-motor associations capable of sustaining early imitation skills through self-exploration. The purpose of our proposed mirror neuron model is to *permit the robot to associate the perceived self-performed action with actions in its own (self) motor repertoire empowering it to understand the perceived movement by taking into account the view-dependency of neurons in both F5 and STS as a probable outcome of their associative connectivity*. Accordingly, it might be the opportunity to see the actions of others from a number of perspectives that endows neurons with the capacity to respond to the sight of actions across different perspectives. The sight of an action could then trigger corresponding motor actions because it activates the same visuo-motor neurons that have been linked to the observer’s motor command during body-babbling.

Furthermore, we will demonstrate that it is feasible to carry out vigorous self-exploration on the basis of matching kinaesthetic experience to visual motion. In our mirror experiment, a humanoid robot stands in front of a mirror (represented through self-image using camera) in order to obtain the associative relationship between his own motor generated actions and (a mirror image of his own) visual body-image. Through self-exploration, the humanoid robot incrementally learns the mapping between body image and corresponding motor actions by standing in front of the mirror, executing actions, and processing the visual images of the body it observes.

### Related Work

The discovery of mirror neurons have led to the development of various computational models [34]. These models can be divided into two groups. The first group discusses the biologically realistic models of mirror neurons dealing directly with modelling the neural circuitry for e.g., MSH [35], MNS-1 [36] and MNS-2 [37], Chain Model [38], Sensori-motor processing model [39]. On the other hand, second group deals with the robotic imitation models associated with the mirror neurons for instance connectionist model [40], developmental model [41], common coding paradigm [42]. However, most these models do not attend to the view-invariant property, but instead presume some sort of visual preprocessing (in STS) which leads to perspective invariance [43]

Meltzoff and Moore [44] present a theoretical model of infant facial imitation based on ‘active intermodal mapping’ (AIM). AIM puts forward an intermodal mechanism for imitation. They assert that imitation is a matching-to-target process. The active nature of the matching process is depicted by the proprioceptive feedback loop. This loop enables infants’ motor operation to be assessed against the observed target. According to this model, the observed and generated acts are coded within a supramodal framework, which facilitates infants to ascertain equivalences amongst their own actions and the ones they observe.

Nagai et al. [45] have proposed a computational model for the development of mirror neurons system through self-other correspondence. The model relies on the notion that as vision develops the robot/infant was able to discriminate between self and other actions. The imitation learning differentiate between the self and others where the robot first separately observes the motions and motor commands of self and others and then map and associate the others motions with the self-motor commands. The model relies on the processing of the visually perceived stimuli through the optical flow detected from robots/infant’s vision. The association between the vision and motor commands is developed through the Hebbian learning. Thus the robot’s vision is limited only to hand movements.

The importance of learning through self-observation have also been pointed out by others. For example, Chaminade et al. [46] proposed the hypothesis that sensorimotor associations for hands and fingers learned from self-observation during motor babbling could be used to bootstrap imitative abilities. Similarly, Kuniyoshi et al. [47] created a humanoid robot that learns to imitate gestural movements by performing self-exploratory sensory motor learning. However, these models are limited only to hand movements and other problems of imitation learning, such as automated motion segmentation and clustering and learning of new motion primitives, have not been addressed.

Saegusa et al. [48] describes a developmental framework for action-driven development for the self and action perception. They hypothesised that the results of actions can lead to identification of dynamically changing body and the environment. The robot develops its perception ability by defining its own body with self-generated actions. This leads to the development of action perception based on observation. The self-perception is generated in the robot through random generating some actions though vision and proprioception. Later the robot develops its body image and motor skills. Based on the visual motion segmentation, the robot identifies its body from the environment using visuomotor correlation between the self-action and object.

Gold and Scassellati [49] have developed a model based on the classsical mirror test, for self-recognition through expecting motion in the visual field utilizing an action perception loop. During experimentation, the robot can only view the limited part of the body which limits the observation of the whole body. The mirror in this experiment is only utilized to differentiate between self and other.

Rebrova et al. [43] developed a MNS model accounting for the view invariant neurons in both STS and F5, however, their model did not describe the development of mirroring property and primitive imitation skills. In this paper we extend the MNS model of Rebrova et al. [43] and develop an imitation system that enables a robot to autonomously learn primitive concepts through self-exploration using body babbling with no a priori knowledge of its motor system or the external environment. In designing an imitation learning system, we have addressed the following fundamental issues of automated motion primitive segment without relying on the a priori information about the kinematic model, incremental learning of motion primitives, visuomotor correspondence and linking the observed self-exploratory action with appropriate motor commands focusing on the actions performed by the demonstrator rather than the view-point from which it is observed.

## Methods

**Fig 2:**
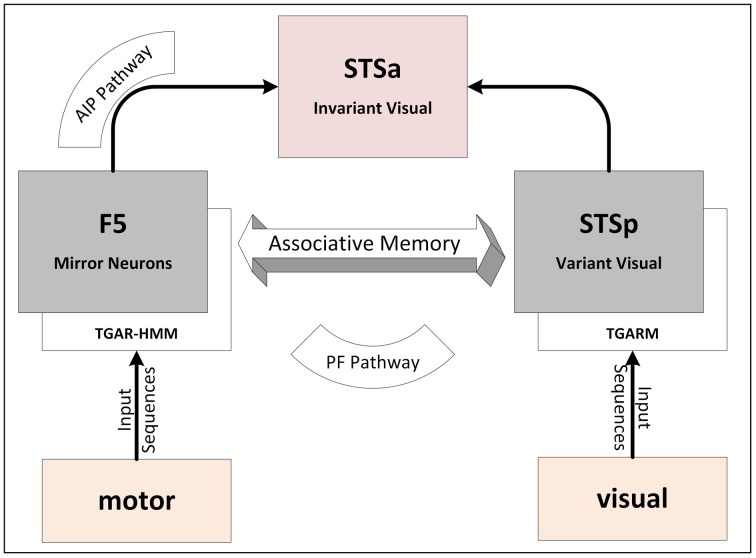
Computational model of the mirror neuron system.

The proposed Mirror Neuron System (MNS) model is adapted from the mirror neuron model presented by Rebrova et al. [43]. Outline of the model is presented in Fig 2. Our computational mirror neuron system for humanoid robot consists of four layers. The first layer (at the bottom) represents the motor and visual information. Motor data, comprised of joint angle values, is acquired from robot sensors whereas the visual sequences are represented by the raw images captured from the robot’s on-board vision sensors (monocular camera). The motor and visual information is then processed to form a high-level representation at the second layer through two modules F5 and STSp, respectively. F5 module is implemented using Topological Gaussian Adaptive Resonance Hidden Markov Model (TGAR-HMM) and the STSp module is implemented as Incremental Kernel Slow Feature Analysis (Inc-KSFA) and Topological Gaussian Adaptive Resonance Map (TGARM).

The mirror neurons represented by the area F5 are connected with representation in STSp through the PF pathway. PF pathway (F5-PF-STSp) forms a layer of neurons in associative network to link motor data from F5 with variant visual data in STSp via the parietal area (PF). This pathway is designed by the Topological Gaussian Adaptive Resonance Associative Memory (TGAR-AM). The topmost layer of computational MNS model is the F5-AIP-STSa pathway, which links the mirror neurons in F5 with invariant representations in STSa. This pathway associates the motor data from F5 with invariant visual representations in STSa.

In order to acquire the lower level motor and visual information and to facilitate the development of early imitative abilities, we have implemented a method for self-exploration of humanoid robot through the use of mirror image. To formulate an apprehension of its own actions, the observer begins by gathering visual perception of its own primitive actions using the mirror image reflection. The robot generates random movements of the body and associates the self-produced actions with its effects perceived through vision. By performing actions in front of the mirror, the observer generates a mapping between the motor commands and the consequent perceived visual changes. This process consisting of self-exploration through mirror image perception is called body babbling.

**Fig 3:**
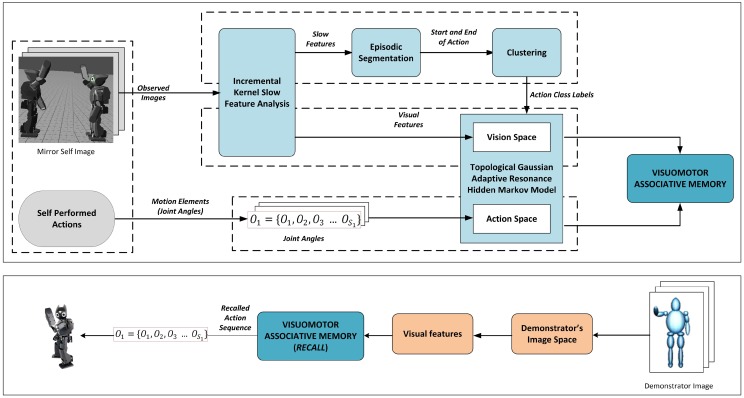
Architecture for incremental imitative learning through self-exploration.

Fig 3 shows the proposed learning architecture for imitation learning through self-exploration. The learning system begins with determining the primitives from continuous movements using on-line segmentation. For this purpose, we have developed an algorithm for automatic segmentation based on the image sequences captured from the robot’s camera. The observed actions are segmented into episodes of different actions determined by the start and end of actions through Incremental Kernel Slow Feature Analysis (Inc-KSFA) algorithm. Inc-KSFA extracts slow varying features from input signals. The variation of slowly changing features is exploited to determine the occurrence of different activities in an incremental fashion. These segmented boundaries of action assists the robot to cluster the observed own actions as primitive actions.

In addition to obtaining the visual images of self performed actions, the joint angle values of the self for different behavioral actions are also acquired as motion elements. These motion elements are mapped onto the action space. The information from the motor representations is processed with F5 module and results in a clustered mappings of illustrations of the same movements. For learning the motion features we proposed a probabilistic incremental learning algorithm called Topological Gaussian Adaptive Resonance Hidden Markov Model (TGAR-HMM). In contrast to conventional HMM, the developed probabilistic learning algorithm is based on incrementally learning spatio-temporal behavioral sequences by developing the graph based structure of the behavior patterns in the form of topological map using Topological Gaussian Adaptive Resonance Map (TGARM). The topological model incrementally updates the number of states and parameters required by the probabilistic model to encode the observed motion elements. This compactly describes the environment as a collection of nodes linked by edges.

Based on segmented data, the learning algorithm is triggered; whenever an action starts, the learning algorithm incrementally encodes the motion elements. The learned motion elements are also labelled using the segments defined during segmentation. In addition, a visual space is developed through TGARM utilizing the features acquired using Inc-KSFA. After generation of these maps independently, the visual space and the action space are connected using associative memory. In order to create an association between the observed visual features (visual space) and self-motor actions (action space), we developed an incremental associative memory architecture called Topological Gaussian Adaptive Resonance Associative Memory (TGAR-AM).

During the learning process, the network first forms mapping from each motor representation onto visual representation from the self-exploratory perspective. Afterwards, the representation of the motor commands is learned to be associated with all possible visual perspectives. This association is developed in order to activate the appropriate motor commands utilizing the visual stimuli. Once the association is developed and learning is complete, we assume that a partner robot comes before the robot performing the similar actions from various perspectives as previously learned by the robot. Based on the sensorimotor association, the robot recalls the corresponding motion pattern from the memory developed during body babbling and performs the actions.

### Motion Primitive Segmentation–Incremental Kernel Slow Feature Analysis

Most of the motion segmentation algorithms for robot learning rely on kinematic structure [50] and very less focus was given to segment motion patterns directly from the vision [51]. When dealing with real-time video sequences, the limit or length of data is not known a priori, therefore, an incremental algorithm is needed. For this purpose, we propose an automatic segmentation algorithm utilizing the image sequences captured from the robot’s vision sensors using Inc-KSFA [52]. In this paper, we study the application of Slow Feature Analyis (SFA) for unsupervised segmentation of motion primitives from a continuous stream of visual data. More precisely, we demonstrate that it is possible to discover the dynamics of motion primitives in an unsupervised manner using Inc-KSFA. Our motivation is based on the close relationship between human perception and SFA [53]. In this paper we used the idea of SFA for video segmentation developed by Liwicki et al. [54]. However, instead of using complex gradient based kernel computed in Krein space [55], we have utilized the method of reproducing kernel in Hilbert space, to reduce computational complexity. Incremental Kernel SFA algorithm does not rely on any predefined training images for processing. It discovers the temporal variations in a video stream online.

The main intuition behind SFA is based on the presumption that information contained in a signal does not change suddenly, but slowly over time [56]. SFA is an unsupervised approach which searches for a set of mappings *g*_*i*_ from *I*–dimensional input data **x**(*t*) = [*x*_1_(*t*), …, *x*_*I*_(*t*)]^*T*^ to generate *J*–dimensional output signal **y**(*t*) = [*y*_1_(*t*), …, *y*_*J*_(*t*)]^*T*^ with components *y*_*j*_(*t*) : = *g*_*j*_(*x*(*t*)) such that *j* ∈ {1, …, *J*}. The optimization problem of SFA is defined by minimizing the temporal variations of the output signal, mathematically:

$$\Delta \left(y_{j}\right):={\langle {\dot{y}}_{j}^{2}\rangle }_{t}\;\text{is}\;\text{minimal}$$ (1)

Under the constraints:

$${\langle y_{j}\rangle }_{t}=0\;\text{(Zero}\;\text{Mean)}$$ (2)

$${\langle y_{j}^{2}\rangle }_{t}=1\;\text{(Unit}\;\text{Variance)}$$ (3)

$$\forall_{i<j},\;{\langle y_{i}y_{j}\rangle }_{t}=0\;\text{(Decorrelation)}$$ (4)

where 〈⋅〉_*t*_ and $\dot{y}$ represent the temporal averaging and the derivative of *y*, respectively. Generally, $\dot{y}$ is measured by the difference between consecutive time steps. The constraints Eqs (2–4) are inserted to prevent constant signals to emerge and avoids trivial solution.

Often, the mapping between the input and output data is assumed to be linear such that the input–output transformation is the weighted sum i.e., *g*(**x**) = **w**^*T*^ **x**(*t*). However, for the real time applications the non-linear input features are expanded through expansion function, *h*(⋅), such that **z**_*i*_ : = *h*(**x**_*i*_). After expansion, the *j* − *th* output signal component is given by $y_{j}\left(t\right)=g_{j}\left(x\left(t\right)\right)=w_{j}^{T}h\left(x\left(t\right)\right)=w_{j}^{T}z\left(t\right)$. Assuming that the signal has unit variance such that the optimization problem is treated as: ${\langle {\dot{y}}_{j}^{2}\rangle }_{t}=w_{j}^{T}{\langle \dot{z}{\dot{z}}^{T}\rangle }_{t}w_{j}$ and ${\langle y_{i}y_{j}\rangle }_{t}=w_{j}^{T}{\langle zz^{T}\rangle }_{t}w_{j}$. Using matrix notations:

$$\min_{W}\;tr\left(W^{T}\dot{Z}{\dot{Z}}^{T}W\right),\;\text{s.t.}\;W^{T}ZZ^{T}W=I$$ (5)

where $\dot{Z}$ represents the temporal derivation matrix, and *tr*(⋅) computes the trace of a matrix. In the first step, the whitening matrix **S** is computed to fulfil the unit variance constraint such that **S**^*T*^ **Z** **Z**^*T*^ **S** = **I**. Then, the directions of least variance in the derivative signals $\dot{Z}$ are found on the derivative covariance matrix $\dot{Z}{\dot{Z}}^{T}$ and represented by an orthogonal matrix **R** to obtain the projection **W** = **SR** which solves Eq (5). The SFA can be represented as:

$$\min_{R}\;tr\left(R^{T}S^{T}\dot{Z}{\dot{Z}}^{T}SR\right),\;\text{s.t.}\;R^{T}S^{T}ZZ^{T}SR=I$$ (6)

In order to find the output of SFA, the Eigen decomposition (ED) of $\dot{\bar{Z}}{\dot{\bar{Z}}}^{T}$ is computed such that $S\dot{\bar{Z}}{\dot{\bar{Z}}}^{T}S=RHR^{T}$. Here $\dot{\bar{Z}}$ represents the centred derivative data matrix. The output of the SFA is given by:

$$c_{j}=R^{T}\left(S^{T}{\bar{z}}_{j}-S^{T}\mu_{\dot{z}}\right)=W^{T}\left({\bar{z}}_{j}-{\dot{\mu }}_{z}\right)$$ (7)

The ordering, in terms of slowness, of the functions **c**_*j*_, is provided by the order of the components in **R** which is governed by the eigenvalues in **H**. The slowest function is related to the smallest eigenvalue and the next larger eigenvalue gives the second slowest function, etc.

#### Incremental Kernel Slow Feature Analysis

Incremental Kernel Slow Feature Analysis updates slow features, incrementally so that it can process new input data by incrementally updating the data mean and whitening projections. Suppose we have a data matrix $X_{A}=\left[{\text{x}}_{1}\;\cdots \;{\text{x}}_{n}\right]\in R^{m\times n}$. In principle we non-linearly map **X**_*A*_ to a higher dimensional space F using the function $\Phi :R^{m}\to F$. Using **Φ**, we transform **X**_*A*_ into **Φ**_*A*_ = [*ϕ*(**x**_1_) ⋯ *ϕ*(**x**_*n*_)]. The map **Φ** is induced by a kernel function *κ*(**a**,**b**) = *ϕ*(**a**) ⋅ *ϕ*(**b**), with $a,b\in R^{m}$ that allows us to evaluate inner products in new space F.

Let the considered mappings be elements of a reproduced kernel Hilbert space (RKHS) [57]. Consider the matrix $K=\Phi_{A}^{T}\Phi_{A}$. By using *κ*(⋅, ⋅), $\Phi_{A}^{T}\Phi_{A}$ can be evaluated without having to perform the mapping Φ since $\Phi_{A}^{T}\Phi_{A}$ contains only dot products between the *ϕ*(**x**_*i*_)s. The optimization problem of SFA can be reformulated as:

$$\min_{R}\;tr\left(R^{T}S^{T}\dot{\bar{K}}{\dot{\bar{K}}}^{T}SR\right),\;\text{s.t.}\;R^{T}S^{T}{\bar{\Phi }}_{A}{\bar{\Phi }}_{A}^{T}SR=I$$ (8)

where $\dot{\bar{K}}$ is the derivative of the centered kernel matrix $\bar{K}$. Let us assume we are given a new data matrix $X_{B}\in R^{m\times i}$ where Φ_*B*_ = *ϕ*(**X**_*B*_). We want to incrementally find the whitening projections and update the slow features to incorporate new data patterns such that the whole information is represented by the concatenation of **Φ**_*A*_ and **Φ**_*B*_ as **X** *_C_* = [**Φ** *_A_* **Φ** *_B_*].

***Zero Mean***: Let ***μ***_*A*_ and ***μ***_*B*_ be the mean of **Φ**_*A*_ and **Φ**_*B*_ respectively with *n*_*A*_ and *n*_*B*_ be the number of data samples contained in **Φ**_*A*_ and **Φ**_*B*_, respectively, we can update the mean *μ*_*C*_ of overall data **X**_*C*_ as [58]:

$$\mu_{C}=\frac{n_{A}}{n_{A}+n_{B}}\underset{\mu_{A}}{\underset{︸}{\Phi_{A}\left(\frac{1}{n_{A}}1_{n_{A}\times 1}\right)}}+\frac{n_{B}}{n_{A}+n_{B}}\underset{\mu_{B}}{\underset{︸}{\Phi_{B}\left(\frac{1}{n_{B}}1_{n_{B}\times 1}\right)}}$$ (9)

Let ${\bar{\Phi }}_{A}$ and ${\bar{\Phi }}_{B}$ be the centered data matrix of **Φ**_*A*_ and **Φ**_*B*_, respectively computed through subtracting the data from its mean to fulfil the zero mean constraint. Similarly, we update the centered matrix of overall data matrix **X**_*C*_ as: ${\bar{X}}_{C}=X_{C}-\mu_{C}$

***Unit Variance and Incremental Whitening***: Consider the matrix $\bar{K}$ such that $\bar{K}={\bar{\Phi }}_{A}^{T}{\bar{\Phi }}_{A}$ and compute the eigenvalue decomposition of $\bar{K}$ as **P** **Λ** **P**^*T*^. Via kernel SVD, we compute the signular value decomposition of $\bar{\Phi }$ as $\left[\bar{\Phi }P\Lambda^{-\frac{1}{2}}\right]\left[\Lambda^{\frac{1}{2}}\right]\left[P^{T}\right]=UDV^{T}$. We want to compute the matrix **S** which whitens the overall data matrix. For this we need to incrementally compute the SVD of the concatenated matrix such that ${\bar{X}}_{C}:\left[{\bar{\Phi }}_{A}\;{\bar{\Phi }}_{B}\right]=U^{\prime }D^{\prime }{V^{\prime }}^{T}$. Let ${\tilde{\Phi }}_{B}$ be the component of Φ_*B*_ orthogonal to **U** and $U^{\prime }=\left[U\;{\tilde{\Phi }}_{B}\right]$ (computed through QR decomposition [59]). The concatenated matrix can be represented in partitioned form as [60]:

$${\bar{X}}_{C}:\left[{\bar{\Phi }}_{A}\;{\bar{\Phi }}_{B}\right]=\;\left[U\;{\tilde{\Phi }}_{B}\right]\underset{\Psi }{\underset{︸}{\left[\begin{array}{cc} D & U^{T}{\bar{\Phi }}_{B}\\ 0 & {\tilde{\Phi }}_{B}\left({\bar{\Phi }}_{B}-{\mathrm{UU}}^{T}{\bar{\Phi }}_{B}\right) \end{array}\right]}}{\left[\begin{array}{cc} V & 0\\ 0 & I \end{array}\right]}^{T}$$ (10)

where **Ψ** is a square matrix of size *k* + *i* where *k* is the number of singular values in **D**. Computing the SVD of $\Psi =\hat{U}\hat{D}{\hat{V}}^{T}$ to diagonalize the matrix and substituting into Eq (10) yields the SVD of ${\bar{X}}_{C}:\left[{\bar{\Phi }}_{A}\;{\bar{\Phi }}_{B}\right]=U^{\prime }D^{\prime }{V^{\prime }}^{T}$.

Since, we are only interested in computing **U**′ and **D**′, **V**′, whose size scales with the number of observed data, need not to be computed. Thus, we only need to calculate the SVD of matrix **Ψ** for the incremental update which is defined as: $U^{\prime }=\left[U\;\;{\tilde{\Phi }}_{B}\right]\hat{U}$ and $\text{and}\;D^{\prime }=\hat{D}$. Therefore, the projection which whitens the signal is computed as **W** = **U**′ **D**′^−1^.

***Slow Feature Update***: Suppose ${\dot{\Phi }}_{A}$, ${\dot{\Phi }}_{B}$ and ${\dot{X}}_{C}$ represents the time derivatives of **Φ**_*A*_, **Φ**_*B*_ and **X**_*C*_, respectively. Let us assume there are ${\dot{n}}_{A}$ samples represented in ${\dot{\Phi }}_{A}$. Similarly, we assume that new dataset ${\dot{\Phi }}_{B}$ consists of ${\dot{n}}_{B}$ samples. We first find the centred data matrix of the newly observed elements represented by ${\dot{\bar{\Phi }}}_{B}$. Thus from [61] we can update the overall data matrix as:

$${\dot{\bar{X}}}_{C}{{\dot{\bar{X}}}_{C}}^{T}={\dot{\bar{\Phi }}}_{A}{{\dot{\bar{\Phi }}}_{A}}^{T}+{\dot{\bar{\Phi }}}_{B}{{\dot{\bar{\Phi }}}_{B}}^{T}+\frac{n_{A}n_{B}}{n_{A}+n_{B}}\left({\dot{\mu }}_{A}-{\dot{\mu }}_{B}\right){\left({\dot{\mu }}_{A}-{\dot{\mu }}_{B}\right)}^{T}$$ (11)

where ${\dot{\mu }}_{A}$ and ${\dot{\mu }}_{B}$ represents the mean of time derivative patterns ${\dot{\Phi }}_{A}$ and ${\dot{\Phi }}_{B}$, respectively. The updated mean of overall data matrix is calculated analogous to Eq 9. Finally we calculate the new feature function by computing the Eigen decomposition of $S^{T}{\dot{\bar{X}}}_{C}{\dot{\bar{X}}}_{C}^{T}S$ as **RHR**^*T*^ which gives our final output. Via the kernel trick, the above process is rendered practicable without explicitly evaluating the mapping *ϕ*(⋅).

#### Episodic Segmentation

We have applied the above explained Inc-KSFA algorithm for the segmentation of actions observed. Eq 1 can be interpreted as the sum of squared Euclidean distance of the slow features computed between consecutive images. Suppose we have data **z**_*i*_, we can define the *δ* as change detected in data after time *t*, as the squared Euclidean difference in slow features among its previous data.

$$\delta_{l_{t}}\left(z_{i}\right)={\left(z_{i}-z_{i-1}\right)}^{T}W_{l_{t}}{W_{l_{t}}}^{T}\left(z_{i}-z_{i-1}\right)$$ (12)

where *l* is the number of utilized slow features.

In order to compare the change between the current frame and the previous time frames, we need to utilize the change of all the previous time steps. Therefore, we need to compute the average change without keeping the previous signals in the memory. The sum of *k* largest eigenvalues in **H** is nearly equivalent to $\mu_{l_{t-1}}=\sum_{1}^{t-1}\delta_{l_{t-1}}\left(z_{i}\right)$. The significant ratio (*ζ*) of the current and mean change is calculated to judge how substantial the change at current step is:

$$\zeta =\frac{\left(t-1\right)\delta_{l_{t-1}}\left(z_{t}\right)}{\mu_{l_{t-1}}}>\tau$$ (13)

where *τ* is the threshold value determined manually. Since the calculation of eigenvalues is done as a part of incremental Kernel SFA, thus, we do not require previous samples. The significance ratio of the current and average change is calculated to judge how substantial the change at current step is. The frames with large variations in the slow features are used to segment the data.

### Topological Gaussian Adaptive Resonance Hidden Markov Model for Incremental Learning

In our proposed architecture, an HMM can be considered as graph whose nodes represent states attainable by the object and whose edges represent transitions between these states. The system is assumed to be at a particular state and to develop stochastically at discrete time steps by following the graph edges according to a transition probability. TGAR-HMM is described as a time evolving HMM with continuous observation variables, where the number of HMM states, structure and probability parameters are updated every time a new observation sequence is available. Structurally, TGAR-HMM are similar to the standard HMMs, besides the fact that the transition structure and the number of states are not constant but vary as more input observation sequences are processed. In addition, the learning algorithm is able to incrementally update the model.

The main idea for developing the proposed probabilistic model is that the structure of the model should consider the spatial structure of the state-space discretization, where the transition among discrete states are only permitted if the corresponding regions are neighbors. Hence, structure learning essentially consists of estimating the suitable space discretization from the observed data and identifying neighboring regions. We have addressed this problem by proposing a topological map using the Topological Gaussian Adaptive Resonance Map (TGARM) algorithm [62].

**Fig 4:**
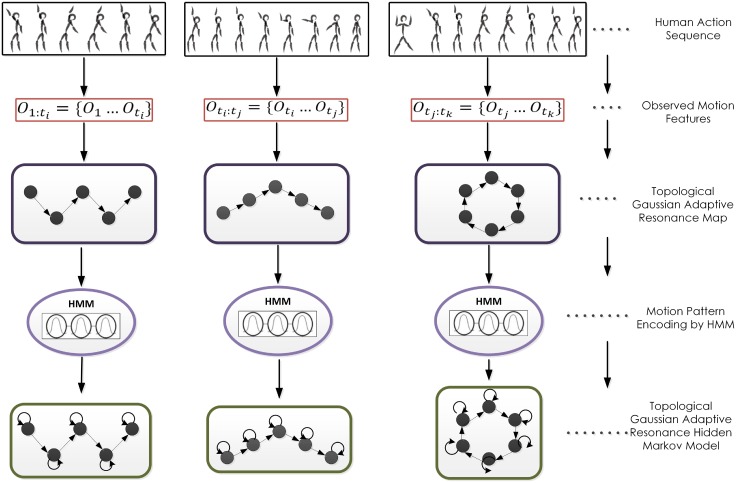
Overview of TGAR-HMM learning architecture. The observed behaviour sequence is first arranged through topological map. This topological map is then used to update the state structure for estimating the optimal number of states and the transition probabilities among these states.

Fig 4 shows the graphical representation of the learning architecture. The observed motion elements (joint angle values) are first organized through topological map consisting of nodes and edges. This map is then used to update the state structure for estimating the optimal number of states and the transition probabilities among the states. The on-line segmentation and learning algorithms together help in grouping different actions in memory.

#### Motion Primitive Modelling

The input to the learning algorithm consists of a series of discrete observation (i.e. joint angle values from sensor reading) describing the motion features. In addition, the observations are arranged in sequences *O*_1:*T*_ = {*O*_1_, …, *O*_*T*_} such that every sequence described the trajectory of action. TGARM inherits the properties of Adaptive Resonance Theory (ART) [63] capable of fast and constructive learning. However, TGARM is able to learn spatio-temporal sequences. The structure grows incrementally by incorporating new knowledge without defiling the previously learned data and adaptively responds to the information acquired from the environment.

**Fig 5:**
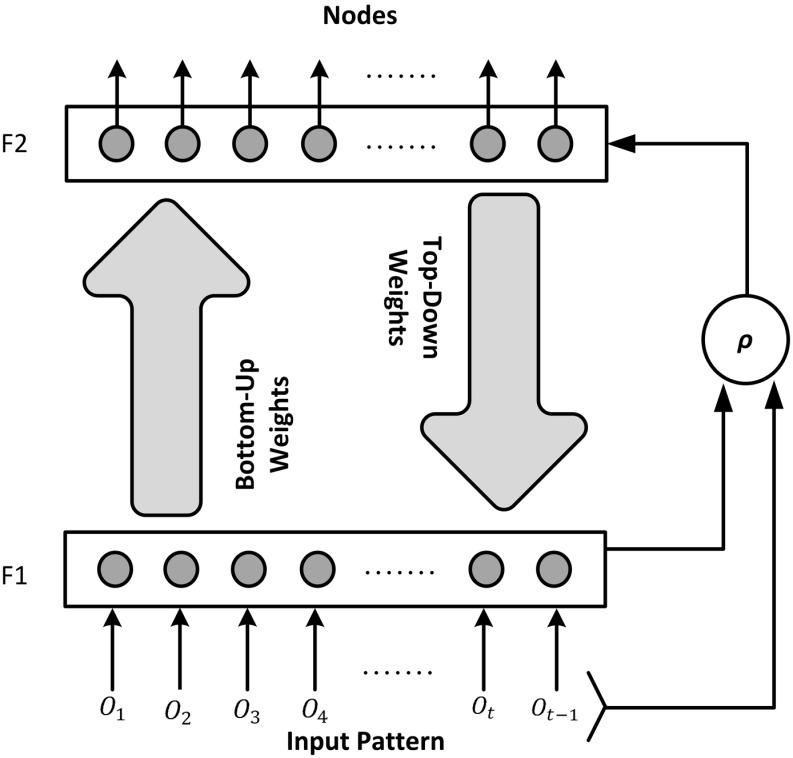
Architecture for creation of topological map.

Fig 5 shows the structure of topological map model. The inputs are received from the robot sensors (for example joint angle values). Each input neuron is connected to the output neurons through the bottom-up weights; conversely, each neuron in the output layer is connected to the input layer through the top-down weights. The bottom-up weights or the activation value provides likelihood that an input pattern is a probable candidate for being a node whereas the matching function provides a confidence measure about the top-down weights. This confidence measure is defined by the vigilance parameter, *ρ*. The output layer creates a topological structure of the input data.

Each node weights are defined by a vector ***ξ***_*j*_,matrix **Γ**_*j*_ and *n*_*j*_ representing its mean, covariance and node count, respectively. The network is initialized with two parameters: the baseline vigilance parameter $\bar{\rho }\in \left(0,1\right)$ and the initial covariance matrix, **Υ**. During learning a winning node *ω*_*J*_ is selected from an input pattern based on the highest probability. The conditional density of **O**_*t*_ given the winning node *j* or the bottom-up input activation value for a node is calculated as:

$$p\left(O_{t}|j\right)=\frac{1}{{\left(2\pi \right)}^{N/2}{\left|\Gamma_{j}\right|}^{1/2}}\exp\left[-\frac{1}{2}{\left(O_{t}-\xi_{j}\right)}^{T}\Gamma_{j}^{-1}\left(O_{t}-\xi_{j}\right)\right]$$ (14)

where *N* is the dimensionality of the input motion patterns.

For each input pattern the activation value is calculated using Eq (14) and the neuron with highest activation value is selected which determines the node with the highest probability: *J* = arg max_*j*_ *p*(**O**_*t*_|*j*). But the node is only allowed to be updated if the vigilance criterion or matching between the given input and the selected winner node is fulfilled. A node *w*_*J*_ passes the vigilance criterion if its match function value exceeds the vigilance parameter value *ρ*, that is if:

$$\exp[-\frac{1}{2}{\left(O_{t}-\xi_{j}\right)}^{T}\Gamma_{j}^{-1}\left(O_{t}-\xi_{j}\right)]\geq \rho$$ (15)

The vigilance is a measure of similarity between the input and the node’s mean relative to its standard deviation. If the winning node fails to pass the vigilance test Eq (15), the current winner node is disqualified and its activation value is reset. Then, the observation pattern is searched for the new winning best-matching neuron. If no satisfactory neuron is found, a new neuron representing the input pattern with *n*_*J*_ = 0 is integrated satisfying the resonance. When the winning neuron, satisfying the vigilance condition representing the input pattern is selected, its parameters i.e. count, mean, and variance is updated using Eqs 16–18.

$$n_{J}=n_{J}+1$$ (16)

$$\xi_{J}=\left(1-\frac{1}{n_{J}}\right)\xi_{J}+\left(\frac{1}{n_{J}}\right)O_{t}$$ (17)

$$\Gamma_{J}=\left(1-\frac{1}{n_{J}}\right)\Gamma_{J}+\left(\frac{1}{n_{J}}\right)\left(O_{t}-\xi_{J}\right){\left(O_{t}-\xi_{J}\right)}^{T}$$ (18)

The algorithm for topological mapping is summarized in Algorithm 1. The learning algorithm grows its neural structure starting with the first node. Each time a behaviour pattern is observed, it is encoded as a Gaussian node in the structure. When the resonating neuron is determined, a lateral connection or an edge is established between the current and previous winner node. This mechanism will provide a stable architecture for establishing a link between the previously learned data and also integrate newly observed data in order to map temporal correlation between them.

**Algorithm 1** Algorithm for Topological Gaussian Adaptive Resonance Map

**Require:**:

 Observation Vector **O**_*t*_

 Initial Covariance Matrix **Υ**

 Baseline Vigilance Parameter $\bar{\rho }$

**Ensure:**:

 Nodes N

 Edges E

1: Input the observation vector **O**_*t*_.

2: **if** There is no node in the network **then**

3: Add **O**_*t*_ in the network as new node. $N\leftarrow N_{i}\cup \left\{O_{t}\right\}$, *n*_*i*_ = 0

4: Update the weights of the node N(n,ξ,Γ) using Eqs (16)–(18)

5: **else**

6: Determine the winner node *ω*_*J*_ from the observation vector using Eq (14)

7: Determine the resonance criterion for the winner node *ω*_*J*_ using Eq (15)

8: **if** calcVig$<\bar{\rho }$ i.e. the observation vector fails the vigilance test **then**

9:  Add as a new node $N\leftarrow N\cup \{O_{t}\}$

10:  Update the weights of the *ω*_*J*_ (winner node) $N(n_{J},\xi_{J},\Gamma_{J})$ using Eqs (16)–(18)

11:  Add edge between the previous winner and current winner nodes $E\leftarrow E\cup \{\left(prevWinner,\omega_{J}\right)\}$

12: **else**

13:  **if** calcVig$>\bar{\rho }$ i.e. the node passed the vigilance test **then**

14:   Reset the winner node and find a new winner from observation vector.

15:   Update the weights of previous winner node.

16:   Obtain the new observation vector **O**_*t*_.

17:   If the learning is not completed, go to Step 6 to process the next observation.

18:  **end if**

19: **end if**

20: **end if**

#### Incremental Learning

After updating the structure of the model, motion patterns are learned through the probabilistic module. This is achieved through Hidden Markov Model. HMM is a doubly stochastic model consists of states which are not directly observed. Each state in the HMM emits an observation as output which infers the most likely dynamical system. Each state is connected by transitions between the states and generates an output pattern. In order to select the appropriate structure of the HMM or selecting the optimum numbers of states, TGARM is employed. After updating the model structure the remaining parameters of HMM, such as transition probability and prior probabilities are updated using EM algorithm.

An HMM is characterized by the following parameters: *State prior probabilities* (*π*_*i*_ = *P*[*s*_0_ = *i*]) represents the prior probability for the corresponding state; *State transition probability matrix* (*a*_*ij*_ = *P*[*s*_*t*+1_ = *j*|*s*_*t*_ = *i*]) represents the probability of transition from state *i* to state *j*; *Observation probability distribution* (*B* = *P*[**O**_*t*_|*s*_*t*_ = *i*]) which is represented by Gaussian function denoted by the parameters *N*(**O**_*t*_|**m**_*i*_,**C**_*i*_), where **m**_*i*_ and **C**_*i*_ is mean vector and the covariance matrix for the *i*-th state in HMM. These HMM parameters are denoted as *λ* = {*π*,**A**,**B**} = {*π*,**A**,**m**,**C**}. Each hidden state in HMM encodes and abstracts an observed motion pattern where a sequence of motion patterns is estimated using the transition between these hidden states.

#### Updating Structure and Parameters of HMM

After updating the topological map, the structure of HMM is also updated based on the added nodes and edges. Corresponding to every node added in the topological map, a state in the HMM is also added. Each added state is initialized with the prior probability *π*_*i*_ = *π*_0_ and self-transition probability *a*_*i*,*i*_ = *a*_0_, where *i* represents the new node. Similarly, for addition of every new node and the new edges (*i*,*j*) connecting these nodes, the transition probabilities are also initialized with state transition probability value *a*_*i*,*j*_ = *a*_0_.

After updating the HMM structure, the parameters of HMM are also updated. The mean and the covariance values related to each Gaussian observation are updated during the structure (topological map) updating process discussed in previous section. The same values are used by the HMM (i.e. **m** = ***ξ***,**C** = **Γ**). However, the remaining parameters such as transition probability and state prior probabilities need to be re-estimated. Traditionally, Baum Welch algorithm [64] which is a type of EM algorithm is used for learning the initial state probability distribution and the sate transition model. Re-estimate the transition probability and state prior probability using Eqs 19 and 20.

$${\bar{a}}_{ij}=\frac{\sum_{t=1}^{T-1}\alpha_{t}\left(i\right)a_{ij}b_{j}\left(O_{t+1}\right)\beta_{t+1}\left(j\right)}{\sum_{t=1}^{T-1}\alpha_{t}\left(i\right)\beta_{t}\left(i\right)}$$ (19)

$${\bar{\pi }}_{i}=\frac{\alpha_{1}\left(i\right)\beta_{1}\left(i\right)}{P(O|s_{i})}$$ (20)

In equations Eqs 19 and 20 the *α*_*i*_ and *β*_*i*_ represent the forward and backward variables [64]. *P*(*O*|*S*_*i*_) in Eq 20 determines the joint observation probability. In order to update the parameters incrementally for new observed data, an incremental learning rule is applied as follows:

$${\bar{a}}_{ij}=\frac{{\bar{a}}_{ij}+\left(N_{p}-1\right)a_{ij}}{N_{p}}$$ (21)

$${\bar{\pi }}_{i}=\frac{{\bar{\pi }}_{i}+\left(N_{p}-1\right)\pi_{i}}{N_{p}}$$ (22)

where *N*_*p*_ is the number input patterns that has been observed until the current time.

### Visuomotor Associative Memory

**Fig 6:**
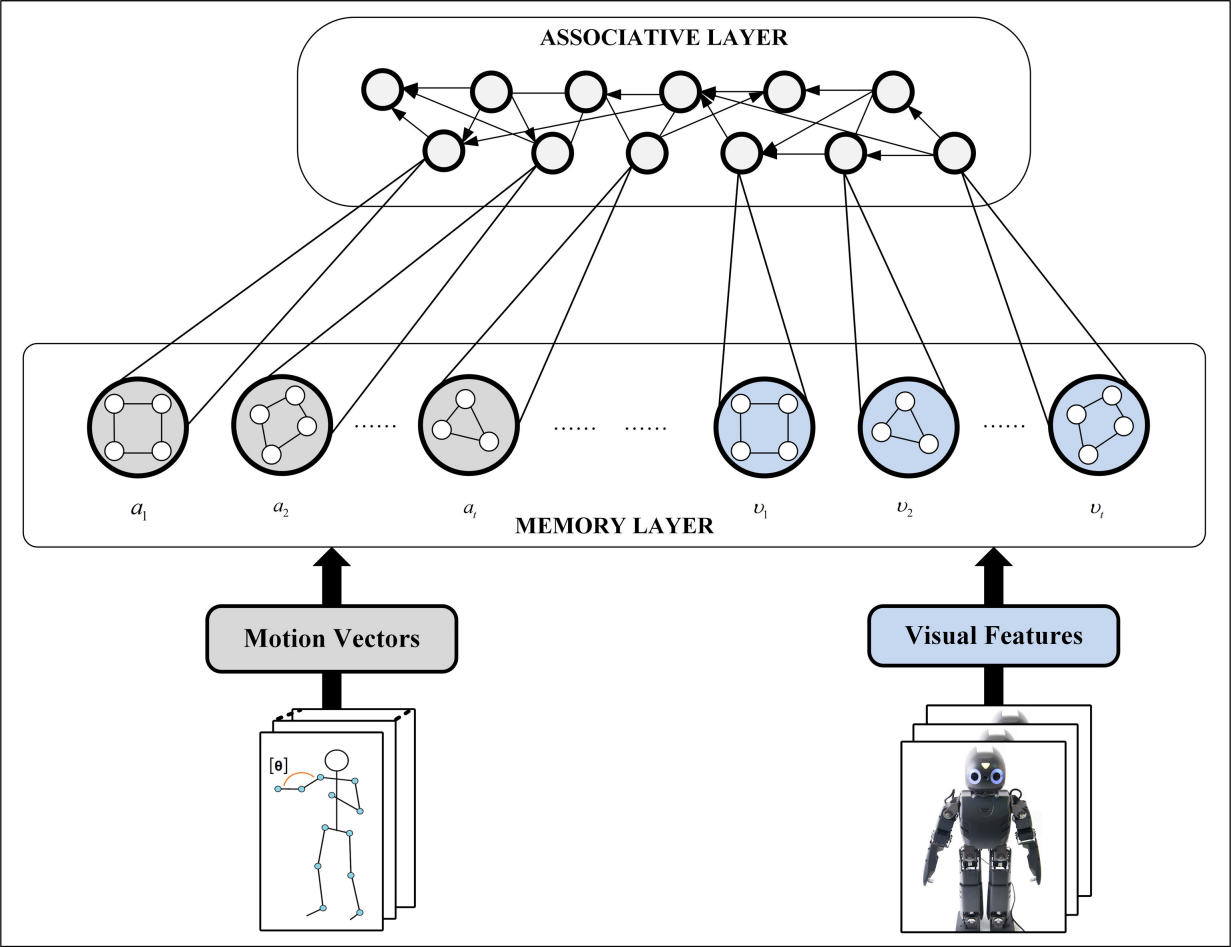
Associative Memory Architecture.

The co-occurrence relationship between motor commands and sensory feedback during body babbling will develop the associations between these two occurrences. Based on this associative relationship, when actions of some other agent are perceived, might lead to an automatic and spontaneous generation of the motor output. We have developed an associative memory, called Topological Gaussian Adaptive Resonance Associative Memory (TGAR-AM), structure using two-layered architecture, namely the memory layer and the association layer (Fig 6). The memory layer encode the received data in the form of a topological structure in an incremental manner, and the association layer formulates the associative relationship between the input patterns. The association between the memorized patterns is developed based on the labels acquired through motion primitive segmentation. According to the labels of these input vectors, the memory layer stores these input patterns as a sub-network. The labels of these sub-networks in the memory layer are passed on to the association layer. Using TGARM, association is developed between the vision (key-vector) and action vectors (response vector). This association between the temporal sequences is represented through the edges between the vision and the action nodes.

The structure of Topological Gaussian Adaptive Resonance Associative Memory (TGAR-AM) is based on TGARM and performs incremental topology representation without calling for a priori definition of the structure and size of the network. For each class of the input feature vectors, we utilized TGARM to represent the distribution of that labelled segment. Based on this theory, the patterns are associated incrementally without defiling the stored knowledge. The proposed associative memory system is able to memorize temporal sequence information as patterns with a consecutive relation.

The main task of behavior generation phase is to find the most likely motion primitive sequence to perform the observed behavioral action. For this purpose the desired behavioral action is presented as an image sequence to the associative memory module. Next, the label of the observed images is estimated using the auto-associative mode and the motion label associated with this observed image is selected. Later, observation sequence from the current observation to the goal observation is generated by most likely path sequence. The TGAR-HMM’s observation-to-observation transition probabilities are used for this purpose to generate the most likely motion primitive sequence.

#### Memory Layer

The memory layer learns input vectors as nodes incrementally, and memorizes the labels of each input vector. When some feature vector is provided as input to the memory layer, if at that point there is no sub-network representing the class label of that input feature vector, then create a new sub-network with the input vector as the first node of the new sub-network and mark this new sub-network with the label of the input feature vector. If there is already a sub-network with the same class name as the input vector, then update the weight vector of the node of the sub-network representing that particular class label. Similar to TGARM, if there is no edge connecting the two nodes, then create and edge linking the two winning nodes. If the label of the input vector does not belong to an existing class in the memory layer, a new network representing the new label is added to the layer. Otherwise, a node is added to the corresponding sub-network. Both the vision vectors and the action vectors are represented by separate sub-networks.

New classes are learned incrementally by adding new subnetworks; for example, learning new patterns belonging to one class is done incrementally by integrating new nodes to an existent subnetwork. The amount of subnetworks is not fixed beforehand, rather determined incrementally based on the number of classes of input patterns. When an input feature vector representing a new class emerges, the memory layer processes the new class without defiling previously learned classes.

#### Association Layer

The association layer builds an association between the vision vectors and the action vectors using their class labels. Suppose we have vision vector $\left(V_{t}\right)$ which we label as visual feature class (*ν*_*t*_) or the key class, similarly, we have motion vectors $\left(A_{t}\right)$ labelled as action feature class (*a*_*t*_) or the response class. Each node in the association layer represents one class and all the nodes are connected through edges—the origin of the edge indicates the visual feature class and the end of the edge points to the corresponding action feature class. During the learning of the association layer, an association paired data consisting of the visual feature and action vector, is utilized as input vectors. First, TGARM algorithm is employed to memorize information of both the vision and the motion feature vectors. The class name of the new class is sent to the association layer. Similar to the memory layer, if the class label of the node in the memory layer does not exist in the association layer, a new node representing the new class label is added to the association layer.

In the association layer, the weight vector of each node is picked out from the corresponding subnetwork of the memory layer. If nodes that represent the vision class (key-class) and action class (response-class) already exist, we link up their nodes with an arrow edge. The origin of the edge indicates the key-class and the end of the edge points to the corresponding response-class. This develops an associative relationship joining the key-class and the response-class.

#### Associative Recall and Behavior Generation

When a key-vector is presented as an input, the associative memory is required to recall the corresponding response vector associated with that particular key from the memory. The recall process employed both auto-associative and hetero-associative mechanism. Behavior generation phase can be described as a two-step problem:

- ***Category Estimation***: Given the visual stimuli observation sequence represented by slow features, the role of category estimation is to determine the label of the unlabelled input visual features. This is accomplished through the auto-associative recall process.
- ***Motion Primitive Sequence Generation***: Given the category of the observed visual stimuli and HMM, the purpose of sequence generation step is to find the associated action category label. This is determined using the hetero-associative mode. After finding the associated category label, the corresponding most likely state sequence for motion generation is estimated.

**Algorithm 2** Algorithm for Auto-associative Recall

1: Input the observation vector $V_{t}$.

2: **for** all the nodes in the Memory Layer. **do**

3: Calculate the weight sum of input vector as:

4: $\vartheta_{i}\left(V_{t}\right)=N_{i}^{T}V_{t}-\frac{1}{2}{∥N_{i}∥}^{2}$ where *N* is the weight of the nodes in the memory layer.

5: **end for**

6: Find: $\vartheta_{k}\left(V_{t}\right)=\max_{\forall nodes}\vartheta_{i}\left(V_{t}\right)$

7: **if** $∥V_{t}{∥}^{2}-2\vartheta_{k}\left(V_{t}\right)>\theta$. **then**

8: OUTPUT: Failed to Recall the memorized pattern.

9: **else**

10: Find the node $V_{t}$ corresponding to the sub-network *ν*_*t*_.

11: **end if**

In the first step the auto-associative mechanism is invoked which recognizes the key vector class resembling the input patterns stored in the memory. The input pattern may be noise polluted. We find the distance between the input vector $\left(V_{t}\right)$ and the weight vector of the stored patterns $\left({N_{i}}^{T}\right)$. If the distance (*ϑ*) Eq (24) between the two vector lies within the Voronoi region, i.e. the distance is larger than the threshold Eq (23), then the memorized pattern is recalled. Otherwise, the system fails to recall. The threshold value is determined by the vigilance parameter (*ρ*) used during TGARM learning.

$$∥V_{t}{∥}^{2}-2\vartheta_{k}\left(V_{t}\right)>\rho$$ (23)

$$\vartheta_{i}\left(V_{t}\right)={N_{i}}^{T}V_{t}-\frac{1}{2}{∥N_{i}∥}^{2}$$ (24)

Once the class of the key-vector *ν*_*t*_ is determined using Algorithm 2, the hetero-associative mechanism is employed to recall the corresponding class label *a*_*t*_. During this process the key-vectors determined in the previous phase are presented to the system as sequence of cues, and the system recalls the appropriate class labels associated with that key vector.

## Experimental Setup

**Fig 7:**
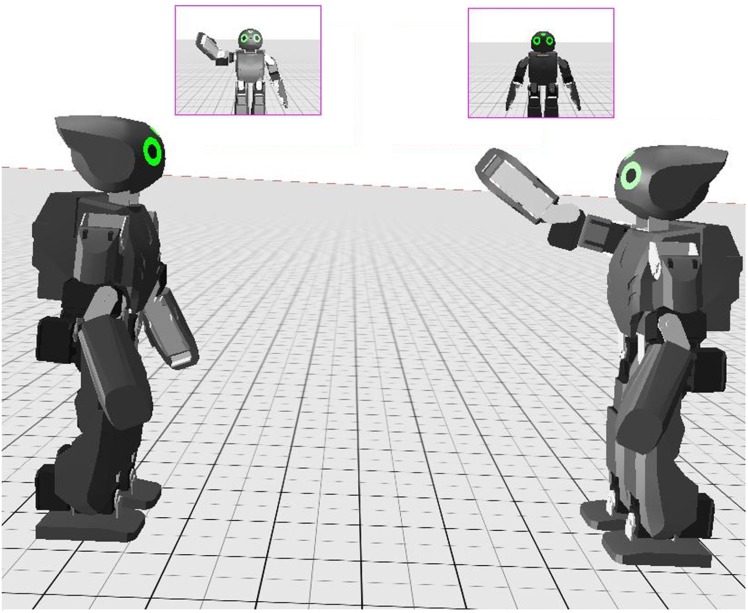
Simulation environment for experimentation consisting of two robots. One acts as a demonstrator (right robot), while the second acts as an observer (left robot).

The assessment of the proposed algorithm was performed through simulation on open humanoid platform DARwIn-OP. For simulation purposes we have used the Webots [65] simulator. The validation of our mirror image based self-learning approach was performed on a test-bed consisting of two DARwIn-OP robots (Fig 7). Just as humans perceive their reflection in the mirror similar to themselves, similarly, in our simulation environment, one robot acts as a demonstrator while the other robot observes these actions as the mirror image reflection of the demonstrator. The algorithm was tested on video sequences of different actions captured by the robot’s camera. At the same time joint angle values of demonstrator are also recorded by the observer which are used for learning the observed action.

## Results

In this section we will discuss the experimental analysis of the proposed approach. During the learning process, the robot performs random arm movements and analyse the image frames grabbed from its head camera. In this case, the robot (self) and its own reflection (interpreted as another robot in simulation environment) moves at the same time during the learning phase. The robot babbled with its motor commands, gathered evidence of the motor commands and corresponding observations, and then learn the relationship between this using (TGAR-AM). During this process, the motor commands interpreted as motion elements (represented through joint angles in radians) are learned through TGAR-HMM.

**Fig 8:**
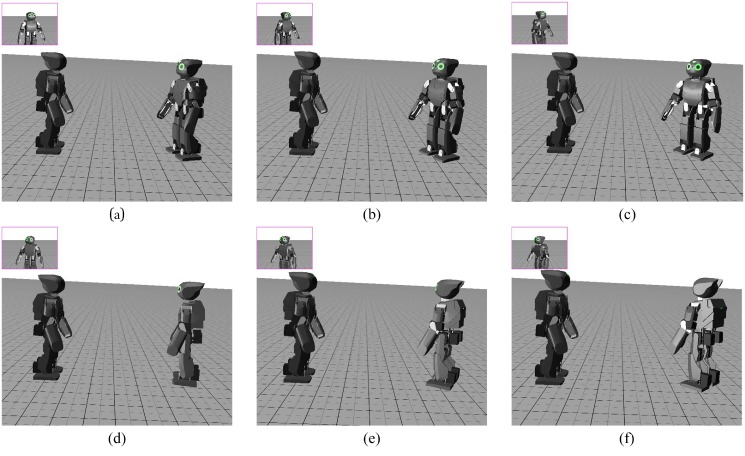
Example of different types of perspectives. (a) v1, (b) v2, (c) v3, (d) v4, (e) v5, (f) v6.

First, the experiments with the self-observing perspective is performed and the association is developed from that perspective symbolizing the PF pathway between STS and F5. The robot associates its own movements with their visual appearance on the basis of TGAR-AM. Directly after this, assuming that the motor patterns are still active, the robot comprehends the same action from a different perspective. Later, the model is trained from different perspectives and the robot performs association with the behavioural actions observed from other different perspectives. To generate visual representations for other perspectives (for e.g. 90°, 180° and 270°), not directly available from simulator, we used self-observed trajectories (0°) and rotated them correspondingly. Fig 8 shows the images of various view perspectives utilized during experimentation.

**Fig 9:**
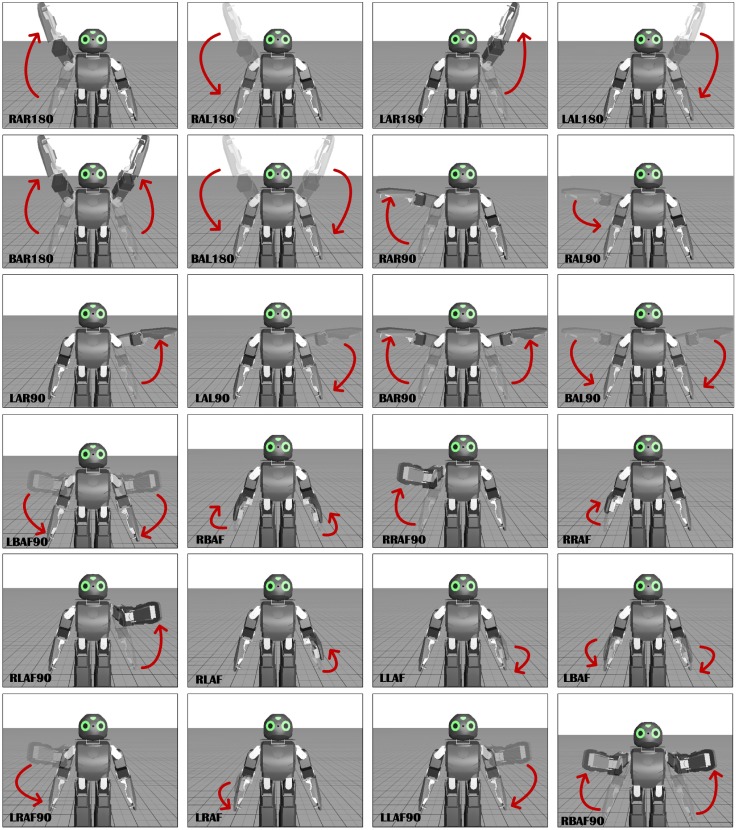
Different samples of actions performed by the robot during experimentation.

The dataset consists of variety of different actions involving upper part of the body. Table 1 summarises the types of actions performed for testing. To assess the efficiency of the proposed model, actions are performed with different repeating intervals, i.e., the sequence of these actions are not fixed and are performed randomly. Some of these actions are performed with a pause between them while others are executed fluidly. These action sequences are performed with varying speeds. Fig 9 shows the visualization of actions performed by the robot. Each image in the figure shows different frames extracted from the action sequences.

**Table 1:** Summary of Different Types of Actions Performed and their identification accuracy.

Motion Description	Label	Segmentation Accuracy [%]	
		Start of Action	End of Action
Right Arm Raise 180deg	RAR180	99.199	98.281
Raise Both Arm Front	RBAF	99.814	99.829
Right Arm Lower 180deg	RAL180	99.316	97.642
Lower Both Arms Front	LBAF	99.791	99.363
Left Arm Raise 180deg	LAR180	99.419	98.835
Raise Left Arm Front 90deg	RLAF90	99.874	99.051
Left Arm Lower 180deg	LAL180	89.611	97.721
Lower Left Arm Front 90deg	LLAF90	89.909	99.263
Both Arms Raise 180deg	BAR180	99.601	88.872
Raise Right Arm Front	RRAF	99.883	99.524
Both Arms Lower 180deg	BAL180	99.633	98.961
Lower Right Arm Front	LRAF	99.832	99.437
Left Arm Raise 90deg	LAR90	99.623	98.843
Raise Right Arm Front 90deg	RRAF90	99.886	99.739
Left Arm Lower 90deg	LAL90	99.376	99.125
Lower Right Arm Front 90deg	LRAF90	99.891	89.444
Right Arm Raise 90deg	RAR90	99.688	96.341
Raise Left Arm Front	RLAF	99.892	99.841
Right Arm Lower 90deg	RAL90	99.809	99.918
Lower Left Arm Front	LLAF	99.924	99.479
Both Arms Raise 90deg	BAR90	99.765	99.496
Raise Both Arms Front 90deg	RBAF90	99.995	99.601
Both Arms Lower 90deg	BAL90	99.791	99.576
Lower Both Arms Front 90deg	LBAF90	99.995	99.476

**Fig 10:**
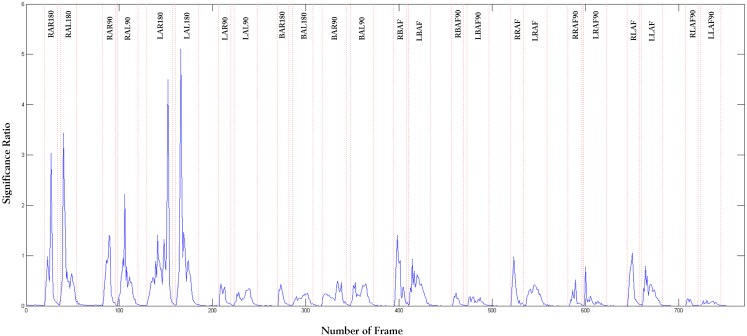
Output of the segmentation algorithm through Incremental Kernel Slow Feature Analysis.

**Fig 11:**
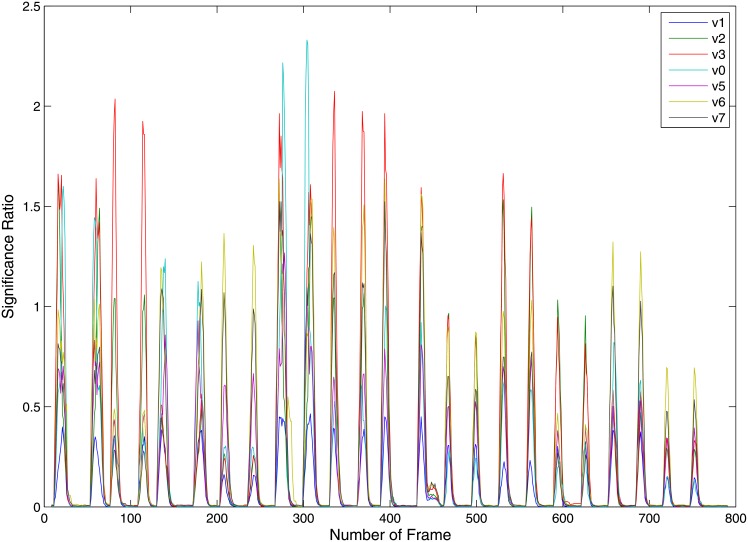
Segmentation through Inc-KSFA for different view perspectives, v1, v2, v3, v4, v5, v6, v7 represents different viewpoints while v0 represents the self-perspective.

The raw image sequences acquired from robot camera are processed for motion primitive segmentation. In order to maintain simplicity, we presume that only one motion primitive is executed at a particular time. Initially, the demonstrator is standing still and no feature points exhibit significant change. As soon as the robot starts moving the joints, change in feature values is recorded and the significant ratio is computed. Based on the significant ratio, the start and end of an action are computed. Fig 10 shows the result of the Inc-KSFA from the self-perspective. We have also tested the segmentation algorithm from different perspectives and summarized the results in Fig 11. These results show that the proposed Inc-KSFA algorithm performs the motion primitive segmentation irrespective of the view or perspective from which the action is observed. The threshold value is selected to be *τ* = 0.0125. Fig 10 shows the change in significant ratio along with the number of frames to segment the observed motion patterns into episodes of action. The segmentation algorithm commences with no a-priori knowledge of the motion patterns and the observed data is being segmented on-line by analysing the incoming data stream irrespective of the view perspective.

**Fig 12:**
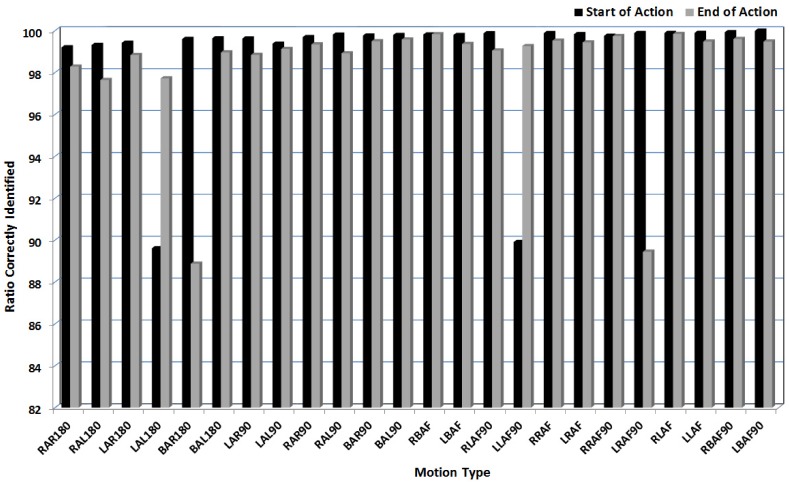
Ratio of average accuracy of segmentation results calculated between joint based segmentation and Incremental Kernel SFA segmentation.

To compare the performance of the proposed algorithm, segmentation is performed based on the change in recorded joint angle values. For joint angle based segmentation, the number of frames are calculated for which there is a change in action. Fig 12 shows the accuracy of segmentation output for different types of actions. The average segmentation ratio is computed for each action performed multiple times and summarized in Table 1. As can be seen from these results, the segmentation of the actions performed produces less error even at the critical points where the actions transit from one motion to other. Since the demonstration is a time-varying (spatio-temporal) representation, the robotic experiments revealed that the recognition or recall is based on retaining the entire sequence of representation units along the trajectory.

The continuous time series data composed of the upper part of the robot and joint angle values (motion elements) are used as input to the learning algorithm. Once the start of an action is detected, TGAR-HMM starts adding the joint angle values as motion primitives in the form of nodes linked with edges to provide temporal correlation. The learning of that particular action is completed when the end of that action is detected. Since behaviour patterns are ordered sequences composed of different atomic actions or motion primitives, each motion element is encoded as a Gaussian. Therefore, we used left-to-right HMM model structure for representing observed motion patterns to allow the data to flow in a sequential order in forward direction of time. In left-to-right HMM the self-transition loop is also allowed. Initially, the topological map is empty, as no motion elements are processed at initialization. Each time a new motion element is observed by the learning algorithm, a corresponding node is added to the topological map. Each node representing the motion element is labelled based on the labels acquired during the motion primitive segmentation. This indicates that the particular segment of motion has been learned as the motion primitive consistent with the added node.

The performance of TGARM greatly depends on the selection of values for the vigilance parameter and initial covariance matrix. For vigilance parameter, the value is chosen to be *ρ* = 0.85, for fast learning and utilizing all the labelled nodes. The reason for selecting a vlaue for the vigilance parameter is to generate the motion pattern as close as possible to the original pattern. This results in selection of optimal number of states or nodes during learning. Similarly, the initial covariance matrix determines the isotropic spread in feature space of a new node’s distribution. The initial covariance matrix is selected in an ad-hoc fashion by trial and error choosing the optimal value for the parameter. The experiments were performed for different values of the covariance matrix and then selecting the value which efficiently generalizes the observed patterns. These values are selected randomly from 0 to 10.The structure of covariance matrix is chosen such that diagonal elements are 0.3 while the remaining vales are zero.

$$CR=\frac{No.\;of\;Samples}{No.\;of\;Nodes}$$ (25)

**Fig 13:**
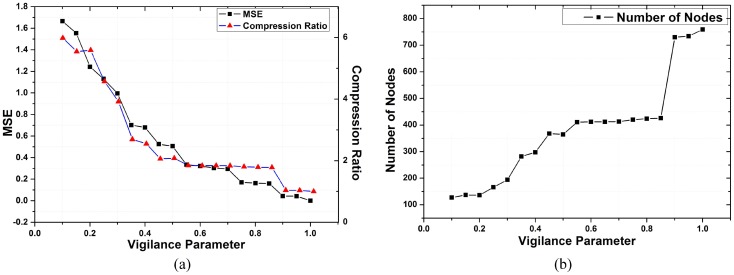
(a) Plot for compression ratio and average mean square error for different values of vigilance parameter. (b) Effect of different values of vigilance parameter on the number of nodes.

Fig 13 shows the effect of selecting different values of vigilance parameter on the compression rate and generalization error. Compression ratio (CR) Eq (25) is determined by dividing the number of data samples in an action pattern and the number of nodes generated by the learning algorithm. As the value of vigilance parameter is increased, the mean square error among the observed and generalized values decreases by adding more number of nodes to the network. For higher values of vigilance parameter, the value of compression ratio is decreased resulting in encoding motion patterns as close as possible to the observed motion.

**Fig 14:**
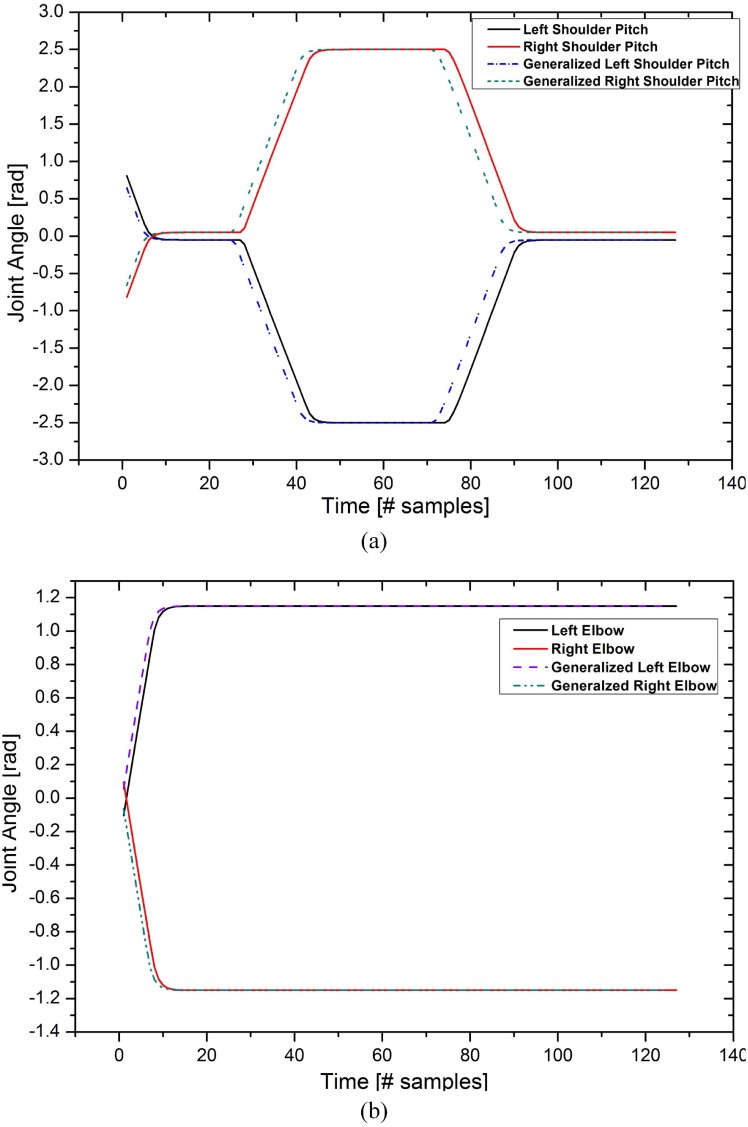
Plot of original and generalized motion patterns for (a)–(b) Raising and Lowering Both Arms 180deg (RBA180–LBA180).

We evaluated the performance of the system using error between the demonstrated and generalized motion to determine the appropriate adapting learned motion. The mean error is used as a metric to evaluate the sustainability of the generalized motion with respect to the demonstrated motion. The mean error is calculated by determining the mean distance of the sample vectors to the nodes created by the learning algorithm. This error metric provides a measure for the evaluation of generalization capability of proposed learning model. Fig 14 shows generalization results for the action of raising both arms (RBA) and lowering both arms (LBA). Since, we are only focused on the upper part of the body for experimentation, therefore, the Fig 14 shows the results for shoulder movements.

**Fig 15:**
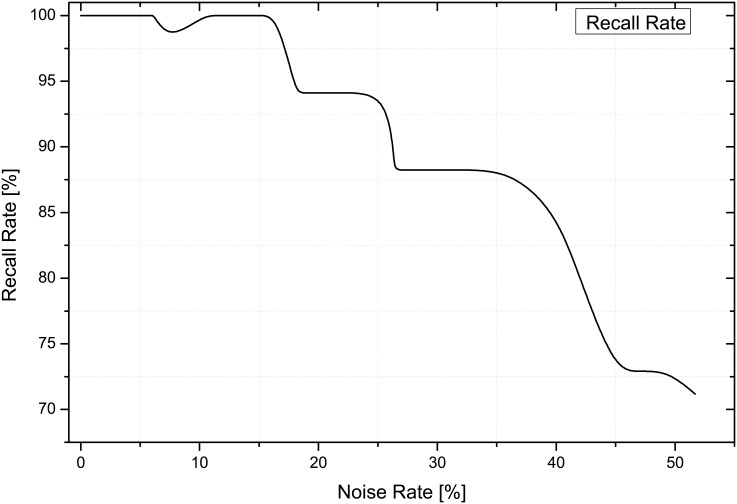
Effect of noise on the recall rate.

During the learning process, the robot babbled with its motor commands, gathered evidence of the motor commands and corresponding observations, and then learn the relationship between these using TGAR-AM. Noise tolerance is a significant function in associative memory. We test the noise tolerance by adding the noises (salt and pepper) on input images sequences randomly. TGAR-AM shows quite high recall rate even if input data contains high noise rate (Fig 15).

During recall process, the robot performs the actions infornt of the partner robot. The sequence of images observed by the robot and the feature values are calculated. Based on the class labels of the acquired image features (category estimation phase), the robot recalls the resulting set of joint-angles (motion primitive sequence generation phase) and the robot arm is able to perform the demonstrated action. Some motion primitives seem to be overlapping in their representation as they are often confused with each other. For example, at the beginning of recognition the RRA is confused with RRAF since the initial position of the joints is almost similar, however, they differ mostly at their final stages as the recognition progresses with more images. Since the demonstration is a time-varying (spatio-temporal) representation, the robotic experiments revealed that the recognition or recall is based on retaining the entire sequence of representation units along the trajectory.

From these experiments we conclude that any visual representation of a specific movement will trigger an appropriate motor command of that particular action, depicting the activity of mirror neuron. The results of the recall rate of the associative memory are summarized in Table 2. The activation of motor information utilizing different perspective visual input can be used to alleviate the process of devising invariant representation of the actions in STSa.

**Table 2:** Result of Associative Recall.

	Number of Experiments	Recall Rate [%]
Self-Perspective	5	100
Perspective v1	5	100
Perspective v2	5	100
Perspective v3	5	99.8
Perspective v4	5	100
Perspective v5	5	100
Perspective v6	5	97

Motion recognition is simply accomplished by recalling the body position from the developed visuomotor memory. The robot observes the demonstration and refers to the visuomotor memory and finds the closest arm joint position on the current visual command. Then, the robot recalls the motion primitive associated with the body position in the memory and moves the arm toward this position. The visuomotor information was memorized when the robot the self-observed demonstration. Note that this motor intention originated only from visuomotor memory, which is the result of self-generated motor exploration. In both the simulation and on the robot the observed interaction is successfully reproduced.

## Discussion and Conclusion

In this paper we present a computational MNS validated on a humanoid platform which revolves around three major concepts related to the origin or development of mirror neurons and primitive imitative abilities. Firstly, we postulate (in agreement with Heyes [17]) that mirror neurons are a by-product of associative learning forged through sensorimotor experience and their actuality is not induced by any evolutionary mechanism [66]. Accordingly, we assumed a link between STS neurons that respond to the vision and F5 neurons which encode the action [67]. The experiments conducted by Nelissen et al. [13] showed that visual information, encoded in the STS, is sent to ventral premotor cortex (F5) along two distinct pathways. One path links the posterior end of the STS with area PFG. The other path associates the anterior part of the STS with F5 via AIP. The first path underlines the action, whereas, the second path accentuates the object/agent of the action. In this sense, the associative memory aids the development of mirror neurons not by pre-wiring the F5-STS connections but by equipping individuals with a propensity to observe an action and associating that specific action with the appropriate self-generated motor commands. Based on this associative connectivity, when an individual observed a similar action, the STS neurons responds to the vision of this particular action by comparing the visual commands which were associated with individual’s past experience. This would triggers an activity in F5 neurons to perform similar action.

Secondly, there has been a great deal of discussion on the role of mirror neurons in imitation learning, however, the discussion on development of primitive imitative abilities is often overlooked, referring instead to the possibility of its innateness. Therefore, we assume that crude sense of self is the prerequisite for social interaction. In order to imitate an observed behaviour, the observer has to recognize the action, but in order to recognize the actions the observer must be able to perform the action [68]. This task was achieved by viewing the development as an incremental process: infants learn new ability on top of the abilities already present [69]. Prior to the emergence of imitation ability, the affordance relations need to be learned and some perceptual-motor associations need to be formed [25]. Learning the affordance relations, by self-directed experience entails the learning process to associate motor commands with corresponding sensory effects. This serves to close the perception-action loop so that the infants can behave accordingly to produce the desired action effects. This is mainly provided through a self-exploration strategy through which the infant explores its own motor capacities, biomechanical constraints, and discovers the possible contingencies between its own body movements and resultant sensory effects.

Thirdly, we presented a MNS based on the empirical discovery showing that in F5 as well as in STS, majority of the neurons are view-dependent. View invariance egressed on the basis of interaction serve for terminal categorical response of the complete system. The neurons in STS are sensitive to viewpoint or visual perspective from which the object is perceived (viewer-centered), but also there are neurons that are invariant to it (view-invariant or object-centered) [14, 70, 71]. This appropriates the invariant neurons to respond to the movement/object irrespective of the observers view-point and render a high-level categorical representation. How these neurons acquire this property is not entirely clear [67], but in monkeys such viewpoint invariance can emerge after experiencing different perspectives of the same three-dimensional object [72].

We presented a developmental framework of computational mirror neuron system that is able to learn by imitation through self-exploration. The purpose of self-exploration presented in the experiments is to empower the robot’s knowledge and motor control ability to develop. The proposed model is based on the assumption that humanoid robot does not have a priori knowledge about itself. It must therefore build a model of the self. The robot first learns about its own body gathering all information by self-exploration through body babbling. We looked into the proposed system’s ability to imitate from a cognitive science point of view instead of engineering perspective in order to acquire knowledge of the possibility for simple imitation capabilities to be associated with self-experience.

We adopted a cognitive science perspective with the hypothesis that imitation of actions can emerge from the intrinsic properties of a neural associative network fed by spontaneous actions and visual feedback of these actions. Self-learning or self-imitation requires a mapping that associates an observed self-motion with the corresponding motor command. Sensory-motor learning through motor babbling has been demonstrated to be efficacious for autonomous humanoid robots for developing an internal representation of the association amongst self and the surrounding environment. We have implemented a simple method for self-recognition on humanoid robot though the use of mirror image. During this stage, the robot generates random movements of the body and associates the action produced by the self with its effects perceived through vision. The visual space consists of its own body image seen in a mirror.

The objective of results reported here was to test whether the ability to imitate could emerge from learning of sensori-motor associations through self-observation. The results obtained can be considered as the developmental steps towards allowing robots to systematically learn how to integrate perception and action through self-experiences much like a human being does, so as to generate adaptive behaviours efficiently and flexibly. The behavior patterns are considered as a sequence of motion primitives [73], atomic parts of a behavioral sequence. For example, if the demonstrator is performing a fighting action, then each motion sequence may correspond to a simple move, such as kick or punch. Before learning, the motion primitives are segmented autonomously by defining the start and end of particular actions. In the later phase, the segmented motion primitives are learned using an approach for continuous learning. As each motion primitive is learned, it is also organized in a topological map, which is incrementally updated to learn the relationship and sequencing of the motion primitives. The algorithm is capable of learning in real-time, during observation of the demonstrator’s motions. The development of a topological structure of the learned motion primitives allows for easier retrieval, and the automatic generation. A visuomotor association is developed between the self-observed images and segmented motion primitives. The main result from the robotic implementation is that this associative network trained by self-observation is capable of action contagion. It exhibits one-shot imitation i.e., without training the motor code corresponding to a new posture presented can be inferred and hence executed.

A key piece of our learning system is the selection of vigilance parameter which effects the performance of system. Although the selection of vigilance is done manually using trial and error method, but the current experiments shows that once the suitable value of vigilance parameter is selected for a articular dataset, it can be efficiently applied to different kinds of motion sequences. However, an improved method for selection or adaptively modifying the value of vigilance parameter will efficiently provide a better generalization performance.

A possible extension of the proposed model is generation of complex behaviours by the combination of various motion primitives [74, 75]. A new behaviour could be created from a combination of two or more motion primitives learned during self-exploration. The observed complex behaviors are decomposed into motion primitives corresponding to the already learned behaviors. The combination is done by recalling the similar actions from the associative memory. For example, the complex action of clapping consists of recalling the motion primitive of raising the arm followed by the motion primitive of lowering the arm in a sequentially continuous manner. Behaviours are generated by forming an abstraction above the motion primitive level.

Future work will focus on implementing full body motion on the humanoid robot, as well as motions involving interaction with the human environment. Currently, the system can only perform the behavioural gestures, therefore, in future we are planning to focus on the issues of view-invariant imitation for complex actions and tasks involving different types of objects.
