# Correction: View-Invariant Visuomotor Processing in Computational Mirror Neuron System for Humanoid

**DOI:** 10.1371/journal.pone.0154898

**Published:** 2016-04-28

**Authors:** Farhan Dawood, Chu Kiong Loo

The following information is missing from the Funding section: This research is supported by UM Grant Challenge Grant GC003A-14HTM.
